# Seroprevalence of Brucellosis, Leptospirosis, and Q Fever among Butchers and Slaughterhouse Workers in South-Eastern Iran

**DOI:** 10.1371/journal.pone.0144953

**Published:** 2016-01-05

**Authors:** Saber Esmaeili, Saied Reza Naddaf, Behzad Pourhossein, Abdolrazagh Hashemi Shahraki, Fahimeh Bagheri Amiri, Mohammad Mehdi Gouya, Ehsan Mostafavi

**Affiliations:** 1 Department of Epidemiology, Pasteur Institute of Iran, Tehran, Iran; 2 National Reference Laboratory for Plague, Tularemia and Q fever, Research Centre for Emerging and Re-emerging Infectious Diseases, Pasteur Institute of Iran, Akanlu, Kabudar Ahang, Hamadan, Iran; 3 Department of Bacteriology, Faculty of Medical Sciences, Tarbiat Modares University, Tehran, Iran; 4 Department of Parasitology, Pasteur Institute of Iran, Tehran, Iran; 5 Department of Virology, School of Public Health, Tehran University of Medical Sciences, Tehran, Iran; 6 Department of Epidemiology, Faculty of Veterinary Medicine, University of Tehran, Tehran, Iran; 7 Centre of Disease Control (CDC), Ministry of Health, Tehran, Iran; Linneaus University, SWEDEN

## Abstract

Zoonotic diseases can be occupational hazards to people who work in close contact with animals or their carcasses. In this cross-sectional study, 190 sera were collected from butchers and slaughterhouse workers in different regions of the Sistan va Baluchestan province, in Iran in 2011. A questionnaire was filled for each participant to document personal and behavioural information. The sera were tested for detection of specific IgG antibodies against brucellosis, leptospirosis, and Q fever (phase I and II) using commercial enzyme-linked immunosorbent assays (ELISA). The seroprevalence of brucellosis was 7.9%, leptospirosis 23.4%, and phase I and II of Q fever were 18.1% and 14.4%, respectively. The seroprevalence of Q fever and leptospirosis, but not brucellosis, varied among regions within the province (*p* = 0.01). Additionally, a significant relationship was found between seropositivity of Q fever and camel slaughtering (*p* = 0.04). Reduced seropositivity rate of brucellosis was associated with use of personal protective equipment (PPE) (*p* = 0.004). This study shows that brucellosis, leptospirosis and Q fever occur among butchers and slaughterhouse workers in this area.

## Introduction

Many zoonotic diseases and human pathogens are occupational hazards faced by individuals who come into close contact with animals or their carcasses. The probability of contact with zoonotic pathogens while working depends upon various factors, such as the health status of the animals, the type of work performed, the frequency of contact with live animals, carcasses and tissues of slaughtered animals, the use of personal and environmental protective measures, and the attitudes and levels of knowledge of the people at risk [1]. Butchers and slaughterhouse workers are at high risk of contracting zoonotic diseases. In Iran, previous studies have identified zoonoses like brucellosis, leptospirosis and Q fever as potential occupational hazards for slaughterhouse workers [2–5].

Brucellosis is an important zoonosis worldwide affecting both livestock and humans; it is listed among the top ten pathogens at the wildlife-livestock interface [6,7]. It can be transmitted to humans through direct contact with infected tissues (especially genital organs and birth products), inhalation of aerosols, and ingestion of raw milk and dairy products from infected animals [8]. Brucellosis in humans is characterized mainly by intermittent fever, with manifestations such as gastrointestinal, cardiovascular, genitourinary, hematopoietic, nervous, skeletal, pulmonary, cutaneous, and ocular involvement [9].

Leptospirosis is a wide spread zoonotic disease that also affects both humans and animals [10]. The etiological agent, *Leptospira* spp., can be transmitted to humans through broken skin or mucous membranes during contact with tissues, body fluids, and organs from infected animals, or by consumption of food or water contaminated with the urine of infected animals [11]. In humans, leptospirosis cause a wide range of symptoms including fever, myalgia, conjunctivitis, jaundice, kidney failure, meningitis, myocarditis, meningoencephalitis and pulmonary haemorrhage with respiratory failure, which sometimes results in death[10]. The disease is an occupational hazard for farmers, sewer workers, miners, dairy and slaughterhouse workers, and fish industry workers [11].

Q fever is also a significant zoonotic disease caused by the rickettsia-like bacterium *Coxiella burnetii* [12]. This disease is considered to be an occupational hazard for livestock handlers, farmers, veterinarians, and butchers and slaughterhouse workers [13]. Livestock such as cattle, sheep and goats are among the main sources of human infection. In animals, Q fever is mostly asymptomatic or subclinical [12,14]. The disease is mainly transmitted to humans through inhalation of infectious agents, consumption of unpasteurized contaminated milk and dairy products, contact with infectious tissues, and, rarely, via tick bites [15]. About 60% of people infected with Q fever are asymptomatic. The symptoms of acute Q fever in humans may include severe headache, prolonged fever, pneumonia, hepatitis, myalgia, arthralgia, cough, cardiac failure and neurological disorders [12]. Patients with chronic Q fever have symptoms such as endocarditis, vascular infection, and fatigue and have a higher likelihood of abortion and stillbirth [15]. Phase I and II antibodies are detectable in patients with chronic Q fever, but antibodies against phase II are indicative of acute Q fever [12].

As butchers and slaughterhouse workers are in close contact with animals or their body fluids and tissues they are at high risk of contracting zoonotic diseases [16]. Imports of large numbers of livestock from eastern neighbouring countries, Afghanistan and Pakistan, to the Sistan va Baluchestan province in Iran [17], and a recent report of brucellosis and Q fever outbreak in Afghanistan [18] prompted us to evaluate the seroprevalence of brucellosis, leptospirosis and Q fever among butchers and slaughterhouse workers in this province. We also evaluated the risk factors related to these diseases among these individuals.

## Materials and Methods

### Study area

**Fig 1:**
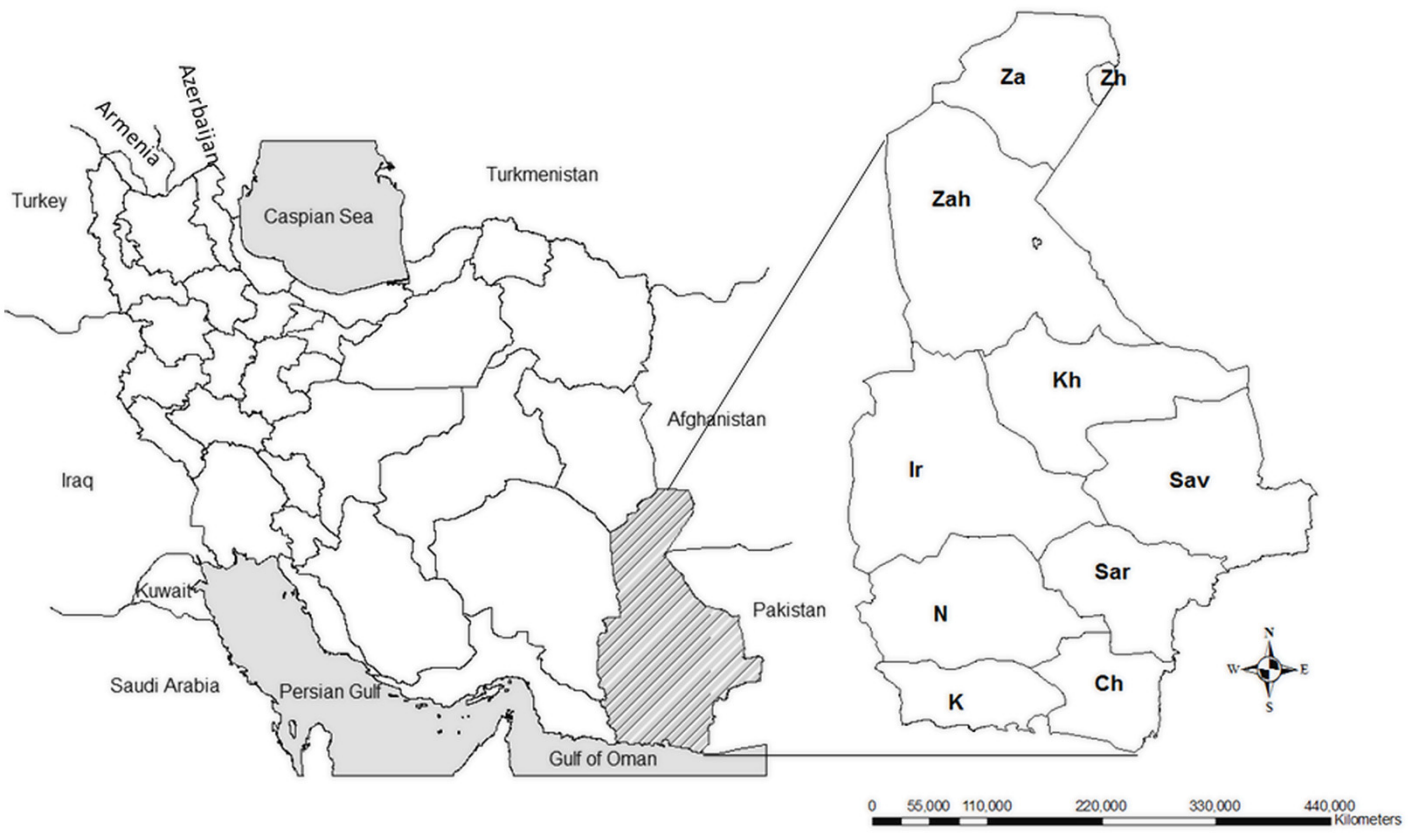
Map of Sistan va Baluchestan province in southeast Iran. Sampling from butchers and slaughterhouse workers was performed in the different counties of Sistan va Baluchestan province including: Zahak (Zh) and Zabol (Za) in the north, Zahedan (Zah), Iranshahr (Ir) and Khash (Kh) in the centre, and Saravan (Sav), Sarbaz (Sar), Nikshahr(N), Konarak (K) and Chabahar (Ch) in the south.

This study was carried out in the Sistan va Baluchestan province in south-eastern Iran in 2011. This province, the largest in the country, covers an area of 187,502 km^2^ and has a population of about 2.5 million people. Sistan va Baluchestan is bordered by the Oman Sea to the south, by Afghanistan and Pakistan in the east, by South Khorasan province to the north, and by the Kerman and Hormozgan provinces in the west (Fig 1). The climate of this province is semi-arid and experiences long, hot summers and short winters.

### Ethical considerations

The ethical committee of the Pasteur Institute of Iran approved the consent procedure, the proposal and protocol of this study, covering all the samples taken (blood), questionnaire and verbal informed consent as most of the participants were either illiterate or had a primary education.

### Samples collection

In this cross-sectional study, blood samples were obtained from butchers and slaughterhouse workers after obtaining their informed consent. All official slaughterhouses in this province were recruited and participants were selected randomly among the employees. Sampling was carried out in slaughterhouses in the north, centre, and south of the province. The inclusion criteria were being over 18 and working as a butcher or slaughterhouse worker for a minimum of 6 months. Information of each participant, including demographic characteristics (age and gender), work history, exposure to risks during work, use of personal protective equipment (PPE) (including mask, gloves, overalls and boots), and their knowledge, attitude, and practices about common zoonotic diseases was collected by means of a researcher-developed questionnaire (Table 1). A 10 mL blood sample was collected from each participant after obtaining informed consent. Sera were kept at -20°C and transferred to the Department of Epidemiology of the Pasteur Institute of Iran (Tehran).

**Table 1:** Analysis of risk factors associated with the seroprevalence of leptospirosis, brucellosis and Q fever among butchers and slaughterhouse workers in south-eastern Iran in 2011.

Risk factor	Leptospirosis			Brucellosis			Q fever		
	No. Tested (% Seropositive)	OR (95% CI)	P- value	No. Tested (% Seropositive)	OR(95% CI)	P-value	No. Tested (% Seropositive)	OR (95% CI)	P-value
**Age (median = 33.5)**									
** ≤33.5 years**	91(21.98)	1.20(0.61, 2.38)	0.60	93(8.6)	0.99(0.91,1.08)	0.78	93(19.4)	1.43(0.76,2.86)	0.31
** >33.5 years**	91(25.27)			93(7.5)			94(25.5)		
**Work History (median = 8)**									
** ≤8 years**	82(21.95)	1.22(0.61, 2.45)	0.58	105(5.7)	2.18(0.74,6.42)	0.14	102(18.6)	1.64(.081,3.32)	0.17
** >8 years**	94(25.53)			77(11.7)			77(27.3)		
**Work Type**									
Slaughtering									
** No**	13(23.08)	1.06(0.29,4.98)	0.97	14(0.0)	1.53(0.23,35.95)	0.61	14(35.7)	0.50(0.16,1.60)	0.32
** Yes**	158(24.05)			162(9.3)			160(21.9)		
Transportation of the remaining livestock									
** No**	129(26.36)	0.56(0.21, 1.34)	0.21	133(7.5)	1.62(0.52,5.03)	0.53	131(23.7)	0.85(0.37,1.97)	0.71
** Yes**	42(16.67)			43(11.6)			43(20.9)		
**Splashing of Animal Secretions**									
Face									
** No**	46(23.91)	0.96(0.44, 2.18)	0.91	47(6.4)	1.34(0.362,4.98)	0.99	47(19.1)	1.30(0.57,2.97)	0.53
** Yes**	138(23.19)			143(8.4)			140(23.6)		
Body									
** No**	36(22.22)	1.09(0.46, 2.76)	0.86	37(8.1)	0.97(0.26,3.64)	0.99	36(25.0)	0.85(0.36,1.98)	0.70
** Yes**	147(31.25)			152(7.9)			150(22.0)		
**Occupational Injury**									
Cutting hand or other organs									
** ≤5 times**	130(23.08)	1.20(0.55,2.55)	0.63	136(8.8)	0.44(0.09,2.04)	0.36	134(19.4)	2.07(0.97,4.341)	0.06
** > 5 times**	49(26.53)			49(4.1)			48(33.3)		
Bite from ectoparasite									
** ≤5 times**	175(22.85)	1.68(0.33,7.07)	0.48	181(8.3)	1.35(0.06,9.21)	0.92	178(21.3)	2.95(0.75,11.51)	0.12
** > 5 time**	9(33.33)			9(0.0)			9(44.4)		
**Contact with Animals**									
Cattle									
** No**	45(31.11)	0.54(0.25,1.19)	0.12	45(8.9)	0.77(0.23,2.59)	0.75	45(26.7)	0.72(0.33,1.56)	0.40
** Yes**	137(19.71)			143(7.0)			140(20.7)		
Sheep & Goat									
** No**	26(15.8)	1.71(0.58,6.11)	0.36	27(3.7)	2.30(0.27,18.21)	0.70	26(11.5)	2.40(0.69,8.46)	0.16
** Yes**	156(28.05)			161(8.1)			159(23.9)		
Camel									
** No**	105(21.90)	1.09(0.53,2.20)	0.81	108(8.3)	0.73(0.24,2.28)	0.60	106(17)	2.01 (0.99,4.09)	0.049
** Yes**	77(23.38)			80(6.2)			79(29.1)		
All 3 group of animals									
** No**	112(20.53)	1.33(0.65,2.72)	0.42	115(8.7)	0.61(0.18,2.02)	0.41	112(17.9)	1.86(0.92,3.74)	0.08
** Yes**	70(25.71)			73(5.5)			73(28.8)		
**Attitude and Practice (Personal Protection)**									
See themselves at risk for zoonotic diseases									
** No**	35(20.00)	1.28(0.53,3.41)	0.60	38(7.9)	0.99(.27,3.71)	0.99	37(10.8)	0.35(.012,1.07)	0.06
** Yes**	148(24.32)			151(7.9)			149(25.5)		
Personal Protection (median = 12)*									
** ≤12**	91(26.37)	1.41(0.71,2.84)	0.33	92(2.2)	0.14(0.03,0.64)	0.004	91(25.3)	1.41(0.70,2.83)	0.34
** >12**	91(19.78)			95(13.7)			93(19.4)		

*Total scores earned by each participant in the use of PPE was considered as the performance of each participant. The median of the performance for all participants was 12.

### Serological tests

#### Detection of brucellosis antibody (IgG)

The commercial enzyme-linked immunosorbent assay (ELISA) kit (IBL, Hamburg, Germany), was used for brucellosis antibody (IgG) detection. Briefly, 1 μL of the sera was diluted 1:100, and after washing with buffer-washing, 100 μL of horseradish peroxidase-conjugated anti-human IgG was added. The mixture was incubated for 30 minutes at room temperature and then treated with tetra methyl benzidine (TMB) for 20 minutes. A *Brucella* antibody-antigen reaction was indicated by a blue coloration. Subsequently, a TMB stop solution was added, and the optical density (OD) of the well was measured at 450 nm by a micro plate reader (ELx808, BioTek Instruments Inc., Winooski, VT, USA). Antibody activities were calculated using a standard curve according to the manufacturer's guidelines.

The positive and borderline sera detected using the ELISA method were confirmed with the standard tube agglutination (STA) test as a gold standard test for brucellosis diagnosis. We used a locally prepared antigen and a STA test produced by the Pasteur Institute of Iran. Sera were serially diluted from 1:20 to 1:1280, mixed with the standard tube agglutination antigen and then incubated at 37°C for approximately 24 hours. Each batch of the test included a positive control and a negative control. Serum titers ≥ 1:80 were considered positive [19,20].

#### Detection of leptospirosis antibody (IgG)

Anti-*Leptospira* IgG antibodies were detected using a commercial ELISA kit (Serion/Verion Co, Germany, Kit number: ESR 125 G) according to the manufacturer's instructions. The plates were read at 405 nm using a micro plate reader (ELx808, BioTek Instruments Inc., Winooski, VT, USA). The cut-off value was calculated on the basis of the standard curve corrected by the mean of the extinction of the standard serum in accordance with the manufacturer's instructions. Quantitative analysis was evaluated for IgG antibodies. A result >30 U/mL was regarded as positive.

#### Detection of *Coxiella burnetii* antibodies (IgG I and II)

IgG antibodies against *C*. *burnetii* were detected using a commercial ELISA kit (Serion/Verion Co., Germany, Kit number ESR 1312 G) according to the manufacturer’s instructions. Phase I and II antibodies were identified in separate assays. The plates were read at 405 nm using a microplate reader (ELx808, BioTek Instruments Inc., Winooski, VT, USA). For phase I antibodies, the sample was considered positive when the serum OD was >10% above the OD cut-off value. For phase II, antibody activities in IU/mL were calculated using a standard curve which was incorporated in the kit following the manufacturer's guidelines.

### Statistical analysis

The data were analysed using SPSS software (Version 16, SPSS Inc, Chicago, Ill). Chi-square, Fisher's exact and logistic regression tests were used to compare the variables during analysis. All results were considered statistically significant if the *p*-value was equal to or less than 0.05, and marginally significant if the p-value was between 0.05 and 0.1.

## Results

In this study, 190 blood samples was taken from butchers and slaughterhouse workers residing in 11 counties of the Sistan va Baluchistan province, including Zahak and Zabol in the north, Iranshahr, Zahedan and Khash in the centre and Chabahar, Sarbaz, Saravan and Konarak in the south of the province. The median (maximum, minimum) age and work experience of participants in this study were 33.5 (18, 86) and 8 (1, 44) years, respectively. All participants were male and 96.8% of them claimed to be satisfied with their occupation. Detailed information about the all participants is shown in S1 Dataset.

In total, 162 (85.3%) participants were directly involved in animal slaughtering, 43 (22.6%) in transportation and handling of animal residues, and 2 (1.1%) only inspected the carcases. A total of 161 (84.7%) participants were in contact with sheep and goats, 143 (75.3%) with cattle including calves, and 80 (42.1%) with camels during their daily activities. Moreover 75.3% of the workers reported a history of being splashed with animal fluids and viscera more than once onto their face and 80.0% on other parts of their bodies. In addition 25.8% of all individuals had a history of cutting their hands or other parts of their bodies at least once during their work, and 17.4% recalled ectoparasite bites within the last year. The obtained data showed that 39.7% of participants did not use any PPE (masks, gloves, overalls and boots), while 22.8% always used it. Also 83.6% of participants had never applied chemical disinfectants to their knives and hands, while 79.9% knew they were at risk of zoonotic infections.

ELISA results revealed that 8.4% of participants were positive and 4.2% at borderline for anti-*Brucella* IgG; the seroprevalence of brucellosis among the participants was 7.9% using the standard tube agglutination test (STAT).

Furthermore, 23.4% and 15.8% of participants were positive and borderline, respectively, for anti-*Leptospira* IgG using ELISA.

Seroprevalence of Q fever antibodies, of phases I and II (IgG) were 18.1% and 14.4%, respectively, and 13.9% and 8.4% of participants had a borderline titre for phase I and phase II antibodies, respectively. The overall seroprevalence of Q fever (participants having antibodies of phase I and/or phase II) was 22.5%.

The highest brucellosis seroprevalence was observed in Chabahar (20.0%) and Nikshahr (20.0%), and the highest Q fever seroprevalence was in Zahak (40.0%) and Iranshahr (42.9%). Leptospirosis seroprevalence was highest in Khash (58.3%) and Zahak (40.0%). The participants from Saravan County in the south of the province had no antibodies to any of the three pathogens (Table 1).

Participants from central and northern regions were 7.26 (OR: 7.26, 95%CI: 1.64, 32.18) (*p* = 0.01) and 10.38 (OR: 10.38, 95%CI: 2.19, 49.13) (*p* = 0.003) times more likely to be seropositive, respectively, for Q fever than those from southern regions. A significant difference in leptospirosis seropositivity was seen between southern (11.4%) and central (32%) regions (OR: 3.66, 95%CI: 1.32, 10.2, *p* = 0.01), but no significant difference was found between southern and northern regions (OR: 2.07, 95%CI: 0.63, 6.76, *p* = 0.23). Brucellosis seroprevalence did not differ significantly among regions (Table 2).

**Table 2:** Seroprevalence of Q fever, brucellosis and leptospirosis among butchers and slaughter workers in Sistan va Baluchestan province according to region and city.

	Leptospirosis			Brucellosis			Q fever		
Region/ County	No tested (% infected)	OR (95% CI)	P value	No. Tested (% infected)	OR (95% CI)	P value	No. Tested (% infected)	OR (95% CI)	P value
**North**	**43(20.9)**	**2.07(0.63, 6.76)**	**0.23**	**44(0.0)**	ND*	**0.999**	**43(32.6)**	**10.38(2.19, 49.13)**	**0.003**
**Zahak**	5(40.0)			5(0.0)			5(40.0)		
**Zabol**	38(18.4)			39(0.0)			38(31.6)		
**Centre**	**97(32.0)**	**3.66(1.32, 10.20)**	**0.01**	**100(11.0)**	**1.3(0.39, 4.32)**	**0.67**	**99(25.3)**	**7.26 (1.64, 32.18)**	**0.01**
**Iranshahr**	13(23.1)			14(7.1)			14(42.9)		
**Zahedan**	72(28.2)			74(12.2)			73(23.3)		
**Khash**	12(58.3)			12(8.3)			12(25.0)		
**South**	**44(11.4)**	**Reference group**		**46(8.7)**	**Reference group**		**45(6.7)**	**Reference group**	
**Chabahar**	10(0.0)			10(20.0)			10(0.0)		
**Sarbaz**	6(16.7)			6(0.0)			5(0.0)		
**Konarak**	10(10.0)			10(0.0)			10(10.0)		
**Saravan**	10(0.0)			10(0.0)			10(0.0)		
**Nikshahr**	8(12.5)			10(20.0)			10(20.0)		
**Total**	**184(23.4)**			**190(7.9)**			**187(22.5)**		

* ND: Not Defined

There were no associations between the age of the investigated persons and the seroprevalence rates of leptospirosis, Q fever or brucellosis. There was, however, a significant association between seroprevalence of Q fever and camels slaughtering (*p* = 0.05, OR: 2.01, 95%CI: 0.99, 4.09). The use of PPE was negatively associated with the seroprevalence of brucellosis (*p* = 0.004, OR: 0.14, 95% CI: 0.03, 0.64). A marginally significant positive correlation was found between seroprevalence of Q fever and having a history of cutting hands (*p* = 0.06, OR: 2.07, 95% CI: 0.97, 4.34) and the attitude of the workers (considering themselves at risk of zoonotic disease, because of their job: *p* = 0.06, OR: 0.35, 95% CI: 0.12, 1.07; Table 1).

Variables such as work history, work type, splashing of animal secretions on face or body and occupational injury were not significantly associated with seroprevalence rates of leptospirosis, brucellosis or Q fever.

## Discussion

In this study we obtained the seroprevalence of antibodies to three zoonotic bacterial diseases among slaughterhouse workers and butchers in the Sistan va Baluchestan province in south-eastern Iran.

Brucellosis is an endemic disease in Iran and has been reported from different parts of the country [21]. In our study, the seroprevalence of brucellosis among butchers and slaughterhouse workers was 7.9%. Different similar studies were conducted among slaughterhouse workers and general population [2, 4, 22–27]. This rate was lower than those obtained from similar studies conducted on butchers and slaughterhouse workers from Khorasan Razavi province in the northeast of Iran (48%) [25], Kerman city in the south of Iran (58.6%) [4], Saudi Arabia (35%) [27], India (25.5%) [28], Pakistan (22%) and (21.7%) [29, 30], and Tanzania (19.5%) [6] and was higher than what was reported from Brazil (4.2%) [31], and South Korea (0.8%) [32] (Table 3). Collectively, these studies indicate that butchers and slaughterhouse workers might face different levels of risk to zoonotic infectious diseases in different areas, possibly due to variation in infection rates among animals, differences in human lifestyle and use of PPE. Taken together, working in a slaughterhouse in Iran seems to be a risk factor for contracting brucellosis, which is further supported by a previous screening among blood donors in the general population in the Bushehr province, southern Iran, that estimated brucellosis seroprevalence to 0.057% [26].

**Table 3:** The comparison of the seroprevalence surveys of brucellosis, leptospirosis and Q fever carried out in different areas among butchers and slaughter workers.

Studies Disease	Region	Number of tested	Seropositivity (%)	*P*-value	OR (%95 CI)	Ref.
**Brucellosis**	This study	190	7.9	Ref.		
	Iran: Gilan	186	9.8	0.54	0.8 (0.38, 1.65)	[22]
	Iran: Shiraz	250	11.7	0.20	0.65 (0.33, 1.25)	[23]
	Iran: Kurdistan	50	12	0.37	0.63 (0.23, 1.86)	[2]
	Iran: Urmia	154	13	0.13	0.57 (0.28, 1.17)	[24]
	Iran: Khorasan Razavi	250	48	<0.001	0.9 (0.05, 0.16)	[25]
	Iran: Kerman	75	58.6	<0.001	0.06 (0.03, 0.12)	[4]
	South-Korea	1482	0.8	<0.001	10.47 (4.79, 23.29)	[32]
	Brazil	551	4.2	0.06	1.97 (0.98, 3.85)	[31]
	Tanzania	41	19.5	0.04	0.36 (0.14, 0.95)	[6]
	Pakistan	260	21.7	<0.001	0.31 (0.17, 0.55)	[30]
	Pakistan	251	22	<0.001	0.31 (0.16, 0.55)	[29]
	India	165	25.5	<0.001	0.3 (0.15, 0.56)	[28]
	Saudi Arabia	269	35	<0.001	0.16 (0.19, 0.28)	[27]
**Leptospirosis**	This study	184	23.4	Ref.		
	Iran: Khoy	30	13	0.22	1.98 (0.69, 6.96)	[36]
	Iran: Zanjan	98	34.7	0.02	0.57 (0.33, 0.98)	[5]
	Iran: Tehran	120	58	<0.001	0.23 (0.14, 0.37)	[37]
	Brazil	150	4	<0.001	7.28 (3.15, 19.38)	[38]
	Mexico	292	8.2	<0.001	3.97 (1.99, 3.90)	[39]
	New Zealand	567	11	<0.001	2.48 (1.60, 3.82)	[40]
	India	20	30	0.25	0.71 (0.26, 2.13)	[41]
**Q Fever**	This study	187	22.5	Ref.		
	Iran: Kurdistan	50	38	0.03	0.47 (0.24, 0.93)	[2]
	Iran: Kerman	75	68	<0.001	0.14 (0.07, 0.25)	[3]
	Brazil	144	29	0.17	0.70 (0.43, 1.16)	[51]
	Turkey	118	50.9	<0.001	0.28 (0.17, 0.46)	[52]

In the present study, 22.8% of participants always used PPE (masks, gloves, overalls and boots) and use of PPE was found to be a protective factor against brucellosis, similar to other studies [6,32]. The accidental splashing of blood and other fluids of infected animals in the vicinity of the mouth and/or injured parts of the body increases the exposure of slaughterhouse workers and butchers to *Brucella* [32]. Previously identified risk factors for the acquisition of brucellosis in Iran include the consumption of fresh cheese, contact with animal skins and consuming undercooked meat or raw milk [33]. Brucellosis is also an occupational hazard for certain professions in health settings, such as veterinary and laboratory personnel’s [33].

The environment and the socioeconomic conditions along the Caspian Sea littoral zone in the north of Iran are more favourable for the survival and transmission of *Leptospira* spp. spirochetes than in other areas of Iran [34,35]. The dry and desert climates in the Sistan va Baluchestan are believed to be less suitable for environmental survival of *Leptospira* spp. spirochetes. This disease can be transmitted to people coming into close contact with animals. Different studies were conducted among slaughterhouse workers and butchers in Iran [5, 36–38]. In our study, the seroprevalence of leptospirosis was 23.4% among butchers and slaughterhouse workers in the province. In other studies carried out on butchers and slaughterhouse workers in Iran, the seroprevalence of leptospirosis was higher in Zanjan province (northwest of Iran)) 34.7% ([5] and in Tehran province (northern Iran)) 58% ([37]. However, in similar studies in other countries, the seroprevalence of leptospirosis was lower than this study: Brazil (4%) [38], Mexico (8.2%) [39] and New Zealand (11%) [40] (Table 3). The comparatively high seropositivity rate in this study is interesting, as no human leptospirosis case has been reported from Sistan va Baluchestan and that none of the seropositive individuals in this study had clinical symptoms registered in their medical history. However, this disease has been reported from South Khorasan and Afghanistan which share borders with Sistan va Baluchestan [42,43]. A study in Pakistan in 2010–2011 revealed a 44% seropositivity rate among veterinarians, pet-owners and livestock holders [44].

The last human case of Q fever in Iran was reported in 1973 [45]. Recently, this infection was reported in livestock in different regions of the country [46–48], and anti-*C*. *burnetii* antibodies were detected in febrile patients in the Kerman province, west of Sistan va Baluchestan [49]. It is known that antibodies against antigens of phase I and II persist for months or years after primary infection. Since the clinical diagnosis is difficult, in most cases, diagnosis of Q fever depends on serological tests [50]. In our study, the seroprevalence rate of phase I and II antibodies in butchers and slaughterhouse workers of Sistan va Baluchestan were 18.1% and 14.4%, respectively.

In other studies carried out on butchers and slaughterhouse workers, the seroprevalence of phase I and II IgG antibodies for Q fever was higher in Kurdistan province, western Iran (38%) [2], Kerman province, south-eastern Iran (68%) [3], Brazil (29%) [51] and Turkey (50.9%) [52] (Table 3). In the present study, the seropositivity of Q fever among butchers and slaughterhouse workers in the central and northern regions of Sistan va Baluchestan was significantly higher than the southern regions of the province, which may be related to the recent human outbreak of Q fever in Afghanistan (Bamyan province in 2011) [18], and/or to larger livestock population in this region and high number of animals imported from Afghanistan. In this study, we observed an association between Q fever seropositivity and camel slaughtering, a finding which is in agreement with other studies reporting Q fever infection in camels in south-eastern Iran [14,53]. Also, in a study in Chad, Africa, camel breeding was a significantly associated with Q fever seropositivity [54].

Apart from occupation, other factors like access to safe water supplies and basic sanitation can contribute to infection risk. The risk of these three bacterial zoonotic diseases to butchers and slaughterhouse workers could have been evaluated more precisely if we had included the general population sera as a control group, which unfortunately was not feasible at the time of the study. Another, drawback was lack of access to the gold standard tests for confirmation of leptospirosis and Q fever.

The current study provided some valuable information on health status of butchers and slaughterhouse workers from south-eastern Iran, which can be useful for health policy makers in their future planning.

## Supporting Information

S1 Dataset Full data set. (XLSX)
